# The expression of prosaposin and its receptors, GRP37 and GPR37L1, are increased in the developing dorsal root ganglion

**DOI:** 10.1371/journal.pone.0255958

**Published:** 2021-08-11

**Authors:** Miho Taniguchi, Hiroaki Nabeka, Kimiko Yamamiya, Md. Sakirul Islam Khan, Tetsuya Shimokawa, Farzana Islam, Takuya Doihara, Hiroyuki Wakisaka, Naoto Kobayashi, Fumihiko Hamada, Seiji Matsuda

**Affiliations:** 1 Department of Anatomy and Embryology, Ehime University Graduate School of Medicine, Toon, Ehime, Japan; 2 Department of Otorhinolaryngology, Ehime University Graduate School of Medicine, Toon, Ehime, Japan; 3 Department of Medical Education Center, Ehime University Graduate School of Medicine, Toon, Ehime, Japan; 4 Department of Human Anatomy, Oita University Faculty of Medicine, Yufu, Oita, Japan; Transilvania University of Brasov: Universitatea Transilvania din Brasov, ROMANIA

## Abstract

Prosaposin (PSAP), a highly conserved glycoprotein, is a precursor of saposins A–D. Accumulating evidence suggests that PSAP is a neurotrophic factor, as well as a regulator of lysosomal enzymes. Recently, the orphan G-protein-coupled receptors GPR37 and GPR37L1 were recognized as PSAP receptors, but their functions have not yet been clarified. In this study, we examined the distribution of PSAP and its receptors in the dorsal root ganglion (DRG) during development using specific antibodies, and showed that PSAP accumulates primarily in lysosomes and is dispersed throughout the cytoplasm of satellite cells. Later, PSAP colocalized with two receptors in satellite cells, and formed a characteristic ring shape approximately 8 weeks after birth, during a period of rapid DRG development. This ring shape, which was only observed around larger neurons, is evidence that several satellite cells are synchronously activated. We found that sortilin, a transporter of a wide variety of intracellular proteins containing PSAP, is strongly localized to the inner side of satellite cells, which contact the neuronal surface. These findings suggest that PSAP and GPR37/GPR37L1 play a role in activating both satellite and nerve cells.

## 1. Introduction

**Fig 1:**
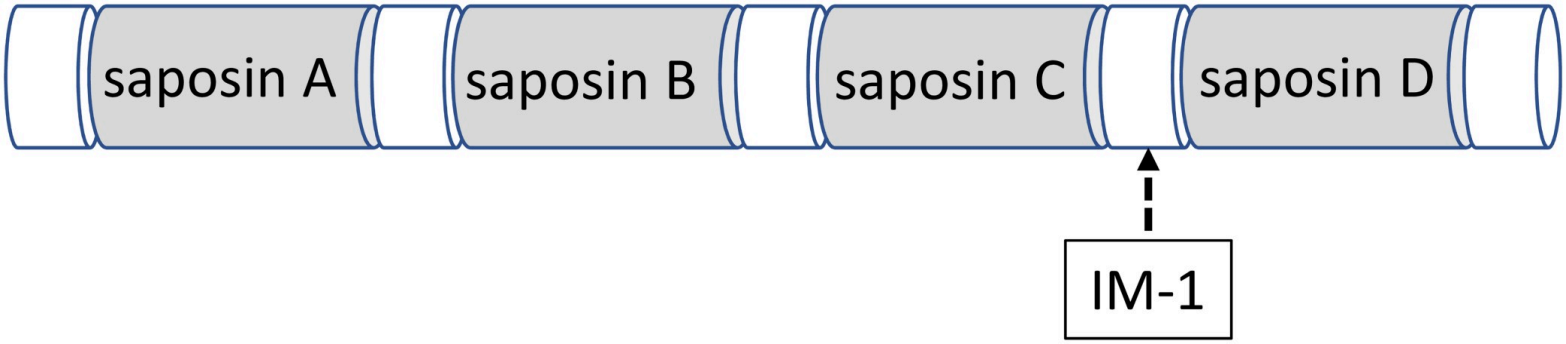
Structure of PSAP and the IM-1 antibody. An anti-rat PSAP-specific antibody (IM-1) was prepared against the proteolytic portion (the intermediate portion between saposins C and D) of PSAP.

Prosaposin (PSAP) is a potent trophic factor and precursor of saposins A–D [1,2] (Fig 1). Saposins are found within lysosomes and activate hydrolysis in a variety of sphingolipids through specific lysosomal hydrolases [3–5]. PSAP has also been identified in various secretory fluids, such as milk, cerebrospinal fluid, and seminal fluid [6–13], suggesting that it serves not only as a precursor of saposins but also as a secretory protein [14]. Finally, PSAP has been shown to act as a potent neurotrophic factor, protecting neural cells against cellular damage [15–20].

The orphan G-protein-coupled receptors GPR37 and GPR37L1 have been defined as PSAP receptors [21]. Both receptors are widely expressed in the brain, where GPR37 has been linked to Parkinson’s disease [22] and GPR37L1 deletion leads to precocious cerebellar development [23]. Neuro- and glio-protection by PSAP is mediated by GPR37 and GPR37L1 [24,25], although the interactions of these receptors with PSAP have yet to be universally acknowledged [26]. We previously showed increased GPR37 and GPR37L1 in astrocytes and microglia, as well as in neurons in the facial nucleus, following transection of the facial nerves [27].

In the present study, using specific antibodies against PSAP, its receptors, and sortilin [28], we investigated the distribution of PSAP and its receptors in the rat dorsal root ganglion (DRG) during development.

## 2. Materials and methods

### 2.1 Animals and tissue preparation

Wistar rats on postnatal days 1 and 3, weeks 1, 2, 4, 6, 8, 12, 16, and 24, and year 2.5 were used in this study. All animals were provided by CLEA Japan (Kyoto, Japan) and housed at a constant temperature (22°C) under a 12:12-h light/dark cycle, and given food and water *ad libitum*. The experiments were conducted in accordance with ARRIVE guidelines and the Guide for Animal Experimentation of the Ehime University School of Medicine, Japan. The protocol was approved by the Animal Care Committee of Ehime University (permit number: 05A261).

Male Wistar rats were anesthetized with medetomidine (0.3 mg/kg), midazolam (4 mg/kg), and butorphanol (5 mg/kg) by intraperitoneal injection and euthanized by cardiac perfusion with physiological saline. Tissues were then fixed in 4% paraformaldehyde and 0.1 M phosphate-buffered saline (PBS, pH 7.4). After fixation, the DRG were dissected, post-fixed in the same solution for 4 h, and then embedded in paraffin using conventional methods. Tissues were sectioned and deparaffinized.

### 2.2 Antibodies against PSAP, GPR37, GPR37L1, and sortilin

The anti-rat PSAP antibody (IM-1) was prepared by Medical and Biological Laboratories (Nakaku, Nagoya, Japan). From the amino acid sequence of rat PSAP, a synthetic oligopeptide corresponding to the proteolytic portion (the intermediate portion between saposins C and D) of PSAP (amino acids 409–434) was used to generate a rabbit polyclonal antibody (IM-1) against rat PSAP (Fig 1). The sequence did not encode a saposin, but was obtained from a PSAP amino acid sequence analysis [29]. Therefore, this IM-1 antibody does not react to saposins; instead, it reacts to PSAP, trisaposin (saposin B-C-D), and disaposin (saposin C-D) (Fig 1).

Specific polyclonal antibodies against two receptors were generated by Eurofins Genomics (Tokyo, Japan) as previously described [29–31]. Briefly, specific antibodies were created by immunising rabbits with synthetic oligopeptides based on rat amino acid protein sequences specific to PSAP (M19936) [32], GPR37 (NP 476549.1) [33], or GPR37L1 (NP 665727.2) [34]. The sequences used were as follows: PSAP, 409-PKEPAPPKQPEEPKQSALRAHVPPQK-434; GPR37, 134-REPTDSQLFRQTSE-147 (#12795V); and GPR37L1, 286-CIMKPSADLPESLYS-300 (#12796V). A commercial antibody against sortilin (EMD Millipore Corp., Billerica, MA, USA) was also used in this study (Fig 9).

### 2.3 Immunohistochemistry of PSAP and its receptors, GPR37 and GPR37L1

Following deparaffinisation and a brief rinse in phosphate-buffered saline (PBS), sections were boiled in 10 mM Tris-EDTA solution (pH 9.0) for 40 min for antigen retrieval. After rinsing with PBS, the sections were incubated in blocking solution containing 5% normal swine serum (NSS), 5% bovine serum albumin (BSA), and 0.25% carrageenan in PBS for 2 h. The sections were processed for immunohistochemistry with primary antibodies against PSAP (IM-1), GPR37, and GPR37L1 at a concentration of 1 μg/ml, and incubated overnight at 4°C. The sections were then rinsed with PBS and incubated with biotinylated anti-rabbit IgG (1:500; Dako, Glostrup, Denmark) for 3 h 30 min at 32°C. After rinsing again with PBS, the sections were incubated with the avidin-biotin complex (ABC) and visualised using a VECTASTAIN ABC kit (Vector Laboratories, Burlingame, CA, USA) for 3 h 30 min at 32°C. Finally, the sections were rinsed again with PBS, and the colour reaction was developed using the diaminobenzidine (DAB) method.

### 2.4 Immunofluorescence of PSAP and its receptors, GPR37 and GPR37L1

Following antigen retrieval, the sections were incubated in blocking solution containing 5% NSS, 5% BSA, and 0.25% carrageenan in PBS for 2 h. The sections were incubated overnight at 4°C with fluorescence-conjugated antibodies. The antibodies used for triple immunofluorescence were Alexa Fluor 594-conjugated anti-PSAP rabbit IgG (red), Alexa Fluor 488-conjugated anti-GPR37 rabbit IgG (green), and Alexa Fluor 405-conjugated anti-GPR37L1 rabbit IgG (blue). The sections were then washed with PBS, mounted in antifade mounting medium (Vector Laboratories) and examined using an A1 confocal microscope (Nikon, Tokyo, Japan).

To assess the immunoreactivity (IR) of the two receptors, antibodies to the receptors were generated with inverted colours: Alexa Fluor 594-conjugated anti-PSAP rabbit IgG (red), Alexa Fluor 405-conjugated anti-GPR37 rabbit IgG (blue), and Alexa Fluor 488-conjugated anti-GPR37L1 rabbit IgG (green) (Fig 6).

### 2.5 Immunofluorescence of PSAP and its receptors, GPR37 and GPR37L1, sortilin, and DAPI

Following antigen retrieval, sections were incubated in blocking solution containing 5% NSS, 5% BSA, and 0.25% carrageenan in PBS for 2 h. The sections were processed for immunohistochemistry with anti-PSAP (IM-1), anti-GPR37 or anti-GPR37L, and anti-sortilin (Abcam, Tokyo, Japan) antibodies at a concentration of 1 μg/ml overnight at 4°C. After washing with PBS, the sections were treated for 5 h at 4°C with Alexa Fluor 594-conjugated goat anti-rabbit IgG (Rockland, Gilbertsville, PA, USA) for detection of PSAP, GPR37, and GPR37L1, and with Alexa Fluor 488-conjugated goat anti-mouse IgG (Rockland) and 4′,6-diamidino-2-phenylindole (DAPI; 1:1000) for detection of sortilin and nuclei, respectively. The sections were then washed with PBS, mounted in antifade mounting medium (Vector Laboratories), and examined using the Nikon A1 confocal microscope.

### 2.6 Immunoelectron microscopy with saposin D antibody

Electron microscopy was performed as previously described [35]. We used anti-saposin D antisera because this antisera can be used for electron microscopy; anti-PSAP (IM-1) cannot be used. Anti-saposin D antisera reacts with both PSAP and saposin D, but immunoblotting of the DRG showed only a band corresponding to PSAP; there was no band for saposin D [35]; thus, the antisera primarily detects PSAP in the DRG. The rats were perfused transcardially with saline followed by 300 ml of 4% paraformaldehyde and 0.1% glutaraldehyde in 0.1 M phosphate buffer. The tissues were cryoprotected and quick-frozen in anhydrous ethanol, rinsed with anhydrous ethanol three times at −30°C; immersed in 30, 60, and 100% LR-White at −30°C; incubated for 3 h at 4°C; and polymerized at 55°C overnight. The thin sections were mounted on nickel grids and incubated on a drop of the blocking solution containing anti-saposin D antiserum. The sections were incubated in blocking solution containing gold-conjugated Fab fragments (15 nm gold anti-rabbit) for 3 h. Sections were rinsed with PBS, fixed in 1% glutaraldehyde, rinsed with distilled water, counterstained, and examined under a transmission electron microscope (H800; Hitachi, Tokyo, Japan).

### 2.7 Validation of the antibodies to GPR37 and GPR37L1

**Fig 2:**
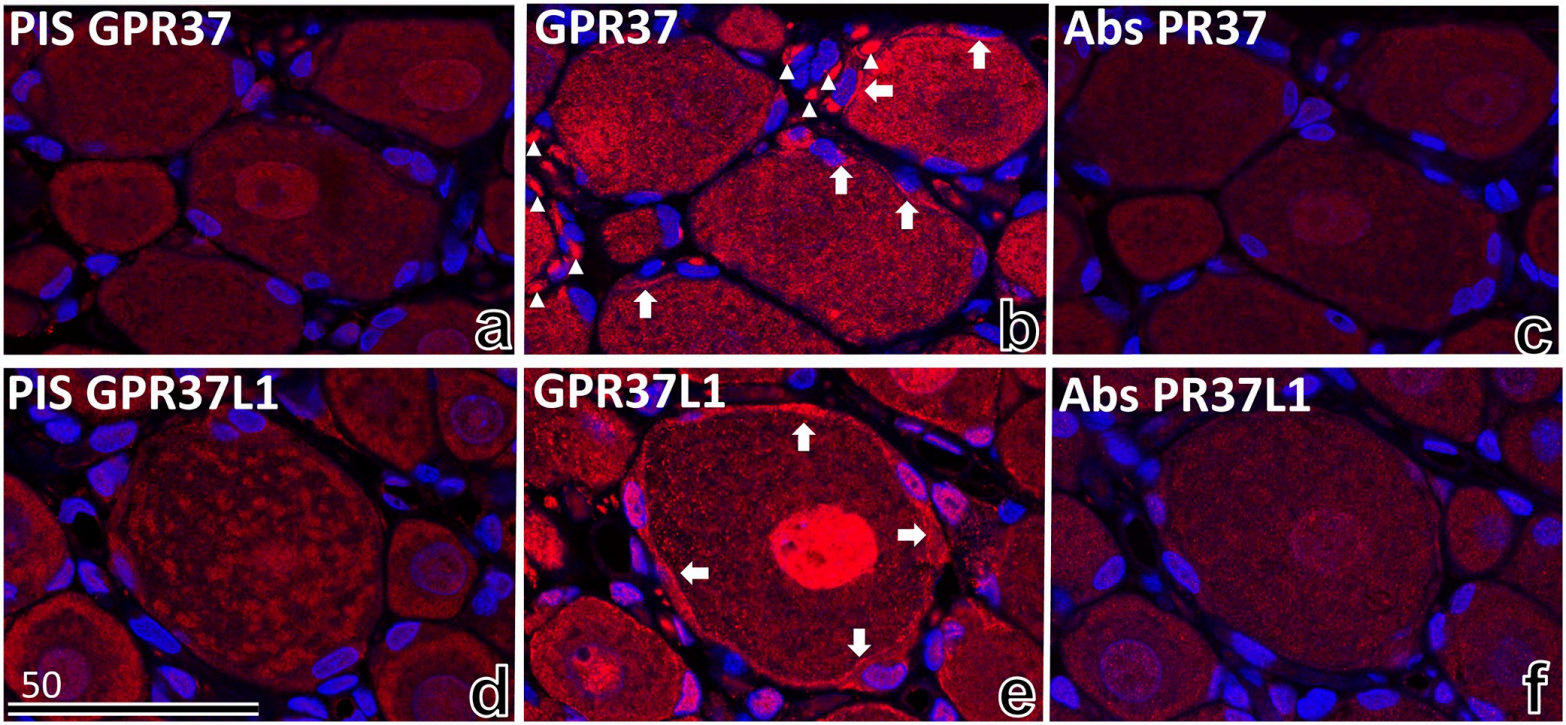
Validation of the antibodies to GPR37 and GPR37L1. Immunofluorescence light micrographs of 8-week-old rat DRG serial sections (a–c, d–f) stained with pre-immune serum (PIS; a, d), anti-GPR37 IgG (b), anti-GPR37L1 IgG (e), anti-GPR37 IgG absorbed with GPR37 antigen (c), or anti-GPR37L1 IgG absorbed with GPR37L1 antigen (f). GPR37 immunoreactivity (GPR37 IR) was observed mainly in the cytoplasm of satellite cells (arrows in b) and Schwan cells (arrowheads in b). GPR37L1 immunoreactivity (GPR37L1 IR) was observed mainly in the cytoplasm of satellite cells (arrows in e) and in the granular structures of neurons and satellite cells (e). Neither GPR37 nor GPR37L1 IR was observed in neighbouring sections stained with pre-immune serum (a, d) or absorbed IgG (c, f). The bars indicate the scale in micrometres.

To validate antibodies against the GPR37 and GPR37L1 receptors, serial sections of DRG from 8-week-old rats were stained with the antibodies. The antibodies were adsorbed with antigen peptide for receptor immunization and with pre-immune serum. For the antibody adsorption, the starting concentration of antibody solution was 10 μg/ml. The antigen peptide solution at a concentration 20 times greater than the original solution was added at the same volume as the antibody solution, and the sample was incubated with rotation overnight at 4°C. The mixture was centrifuged, and the supernatant was used as the antibody solution. The final antibody solution was 1μg/ml and pre-immune serums were diluted 1000 times. Immunofluorescence light micrographs of adjacent tissue sections were used for comparison (Fig 2).

## 3. Results

### 3.1 Validation of the antibodies to GPR37 and GPR37L1

Immunofluorescence light micrographs of rat DRG at 8 weeks of age were stained with pre-immune serum (Fig 2a and 2d), anti-GPR37 IgG (Fig 2b), anti-GPR37L1 IgG (Fig 2e), absorbed anti-GPR37 IgG (Fig 2c), and absorbed anti-GPR37L1 IgG (Fig 2f). GPR37 immunoreactivity (GPR37 IR) was observed mainly in the cytoplasm of satellite cells and Schwan cells. GPR37L1 immunoreactivity (GPR37L1 IR) was high in the cytoplasm of satellite cells but low in the granular structures of neurons and satellite cells (Fig 2e). Neither GPR37 nor GPR37L1 IR was observed in adjacent tissue sections stained with pre-immune serum (Fig 2a and 2d) or absorbed IgG (Fig 2c and 2f). GPR37L1 IR was observed mainly in satellite cells, whereas GPR37 IR was observed in satellite cells and Schwann cells within the perinuclear area (Fig 2b and 2e).

### 3.2 Immunohistochemistry of PSAP and its receptors, GPR37 and GPR37L1

**Fig 3:**
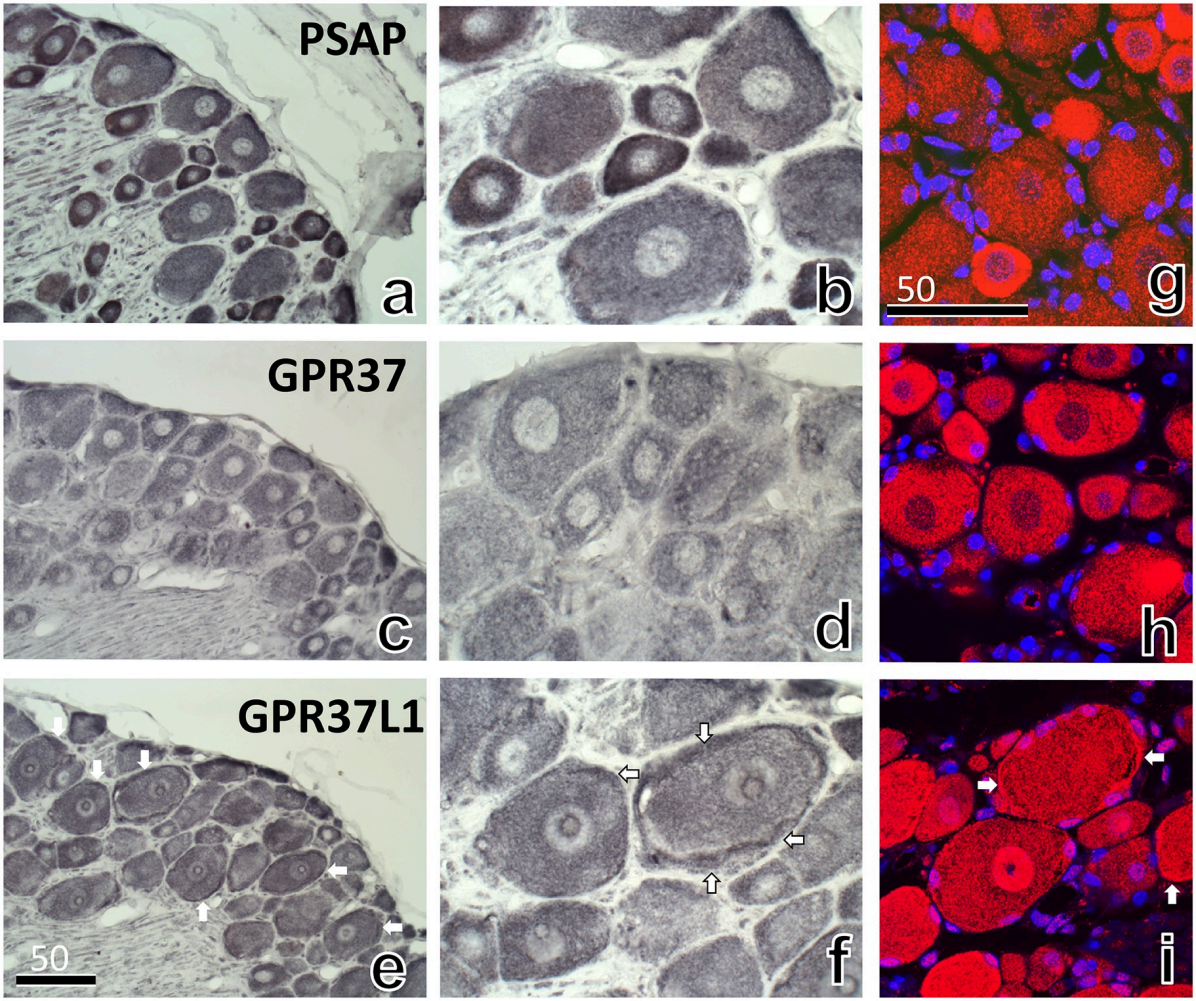
DAB immunohistochemical analyses (a–f) and immunofluorescence (g–i) with antibodies against PSAP (IM-1) (a, b, g), GPR37 (c, d, h), and GPR37L1(e, f, i) in the DRG of rats at 8 weeks after birth. (b, d, and f) Higher magnification images of a, c, and e, respectively. Intense granular PSAP reactions were observed in small neurons, and large dot-like structures were observed in large neurons (b). Similar reactions were shown by immunofluorescence (g). GPR37 exhibited dot-like reactions that were not as strong as those observed with PSAP. GPR37L1 reactivity was similar to that of GPR37 in neurons, and was strong in satellite cells (arrows in e, f, and i), especially those around larger neurons. The numbers on the bars indicate the scale in μm.

To clarify the distribution of PSAP and its receptors, GPR37 and GPR37L1, we first performed DAB immunohistochemistry in the DRG of 8-week-old rats. A strong PSAP signal was observed in nerve cells, especially in small nerve cells (Fig 3a and 3b). GPR37 was weakly (Fig 3c and 3d), and GPR37L1 strongly, distributed in satellite cells around nerve cells (Fig 3e and 3f). Immunofluorescence staining with DAPI and antibodies against PSAP, GPR37, and GPR37L1 showed similar results (Fig 3g–3i).

### 3.3 Immunoelectron microscopy with saposin D antibody

**Fig 4:**
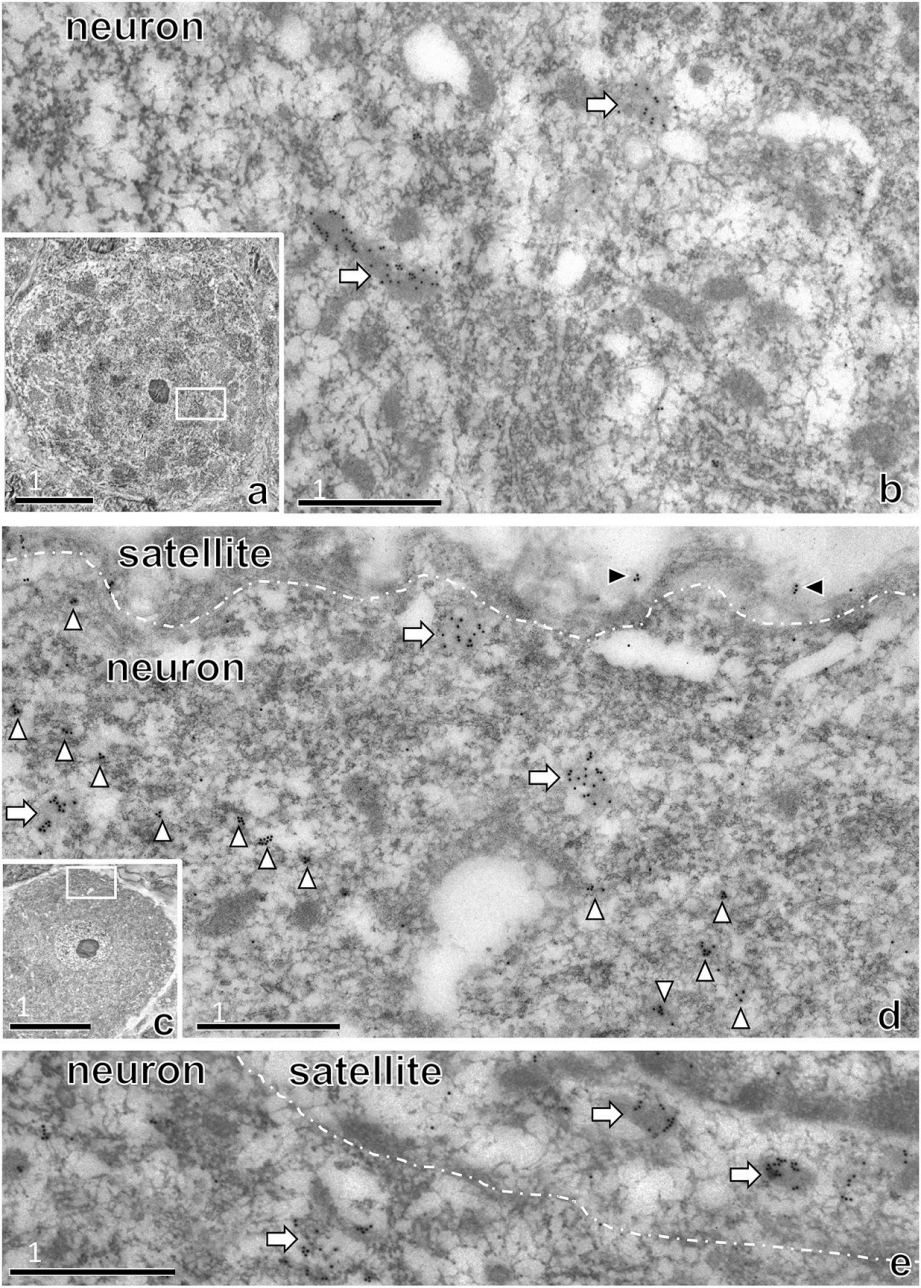
Immunoelectron micrographs of the DRG of rats at 8 weeks after birth stained with anti-saposin D antibody, which also reacts with PSAP. Many immunogold particles (15 nm) were observed in lysosome-like structures in larger neurons (a, b). In smaller neurons (c, d), immunogold particles were observed in many small lysosomes (white arrowheads) and the basal lamina of satellite cells (black arrowheads in d) as well as larger lysosomes (arrows). Many immunogold particles were also observed in the lysosome-like structures of satellite cells (arrows in e). The numbers on the bars indicate the scale in μm.

As observed in the light micrographs shown in Fig 3, immunoelectron micrographs showed many saposin D-positive large lysosome-like structures in larger neurons (Fig 4a and 4b). By contrast, in the smaller neurons, small and large lysosome-like structures were stained with anti-saposin D antibody (Fig 4a and 4b). Immunogold particles were occasionally observed in the basement membrane of satellite cells (Fig 4d). Some lysosomes in satellite cells were also saposin D-positive (Fig 4e).

### 3.4 Triple immunostaining with antibodies against PSAP, GPR37, and GPR37L1

**Fig 5:**
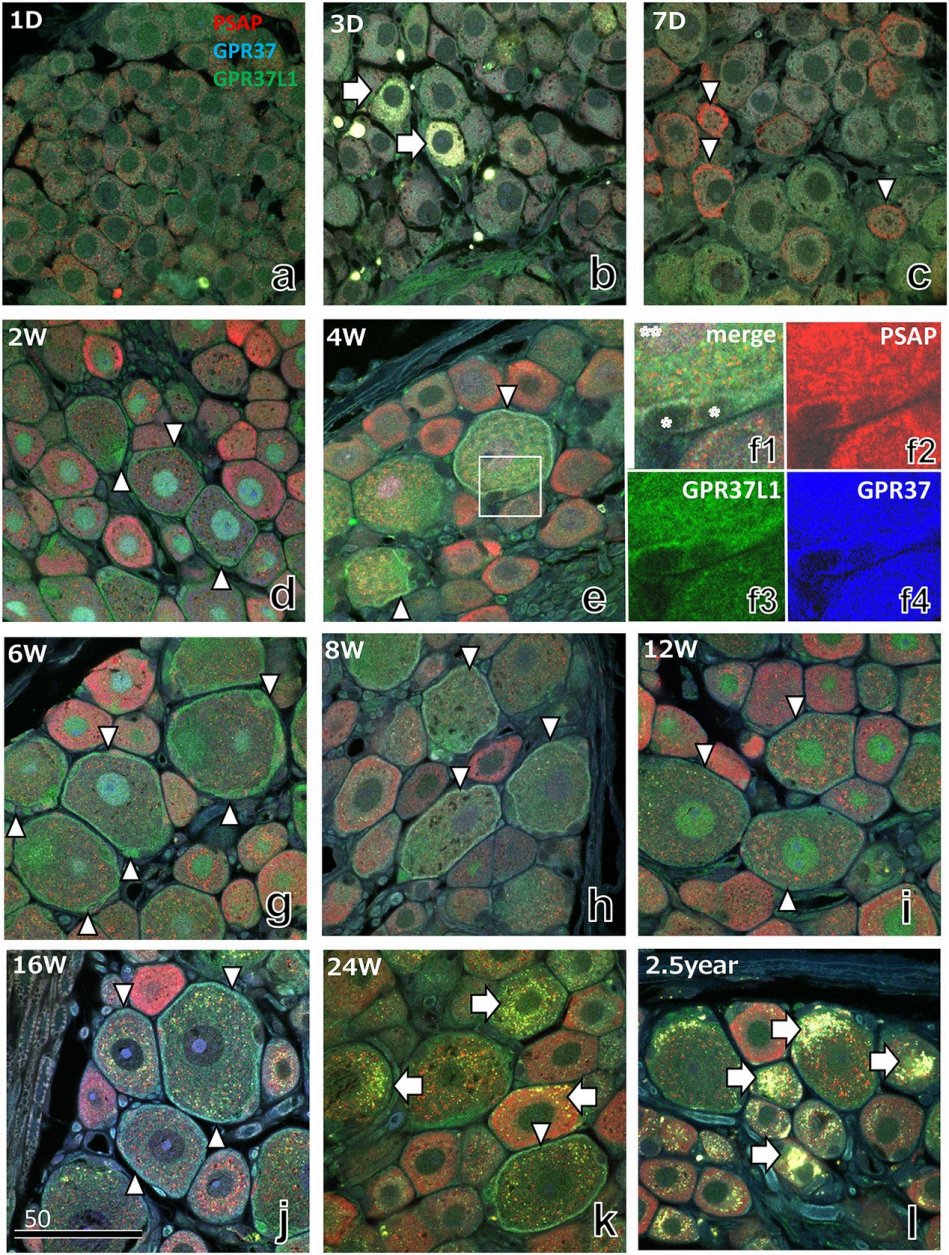
Immunofluorescence light micrographs of the rat DRG from day 1 to 2.5 years after birth stained with anti-PSAP (IM-1, red), anti-GPR37 (blue), and anti-GPR37L1 (green). Before week 1 (a–c), staining was weak, except for some small and damaged neurons (arrowheads in c and white arrows in b, respectively). From 2–12 weeks (d–i), some satellite cells showed strong staining of GPR37L1 (arrowheads in d–k). The area in the rectangle in Fig 5e is shown in higher magnification view (f1–f4). After 16 weeks, these cells were rare. Asterisks indicate the nuclei of satellite cells. Arrows in Fig 5k and 5l indicate lipofuscin granules with strong fluorescence. The numbers on the bars indicate the scale in μm.

The developmental changes in PSAP and its receptors in the DRG from postnatal day 1 to 2.5 years were observed with triple immunostaining using antibodies against PSAP (IM-1), GPR37, and GPR37L1 (Fig 5). On postnatal days 1 and 3, all IRs were weak (Fig 5a and 5b), and on postnatal day 7, moderate PSAP-IR was observed in small neurons (Fig 5c). At postnatal week 2, strong PSAP-IR was observed in small neurons, and IR of receptors was observed in satellite cells around larger neurons (Fig 5d).

At postnatal week 4, IR of PSAP and its receptors was clearly observed (Fig 5e and 5f). The distribution of PSAP-IR differed according to nerve cell size; PSAP-IR was distributed mainly in lysosome-like organelles in large nerve cells, but was distributed in fine organelles in small nerve cells, as demonstrated by DAB staining and immunoelectron microscopy (Figs 3, 4 and 7b and 7f). GPR37-IR and GPR37L1-IR were distributed in satellite cells, especially in those around large nerve cells (Fig 5d–5j). PSAP-IR was observed at all stages studied, but the IR of receptors decreased after 16 weeks, with a peak at 12 weeks (Fig 5j–5l). Strong fluorescence of damaged neurons and lipofuscin granules were observed at postnatal day 3 (arrows in Fig 5b), week 24, and 2.5 years (arrows in Fig 5k and 5l).

**Fig 6:**
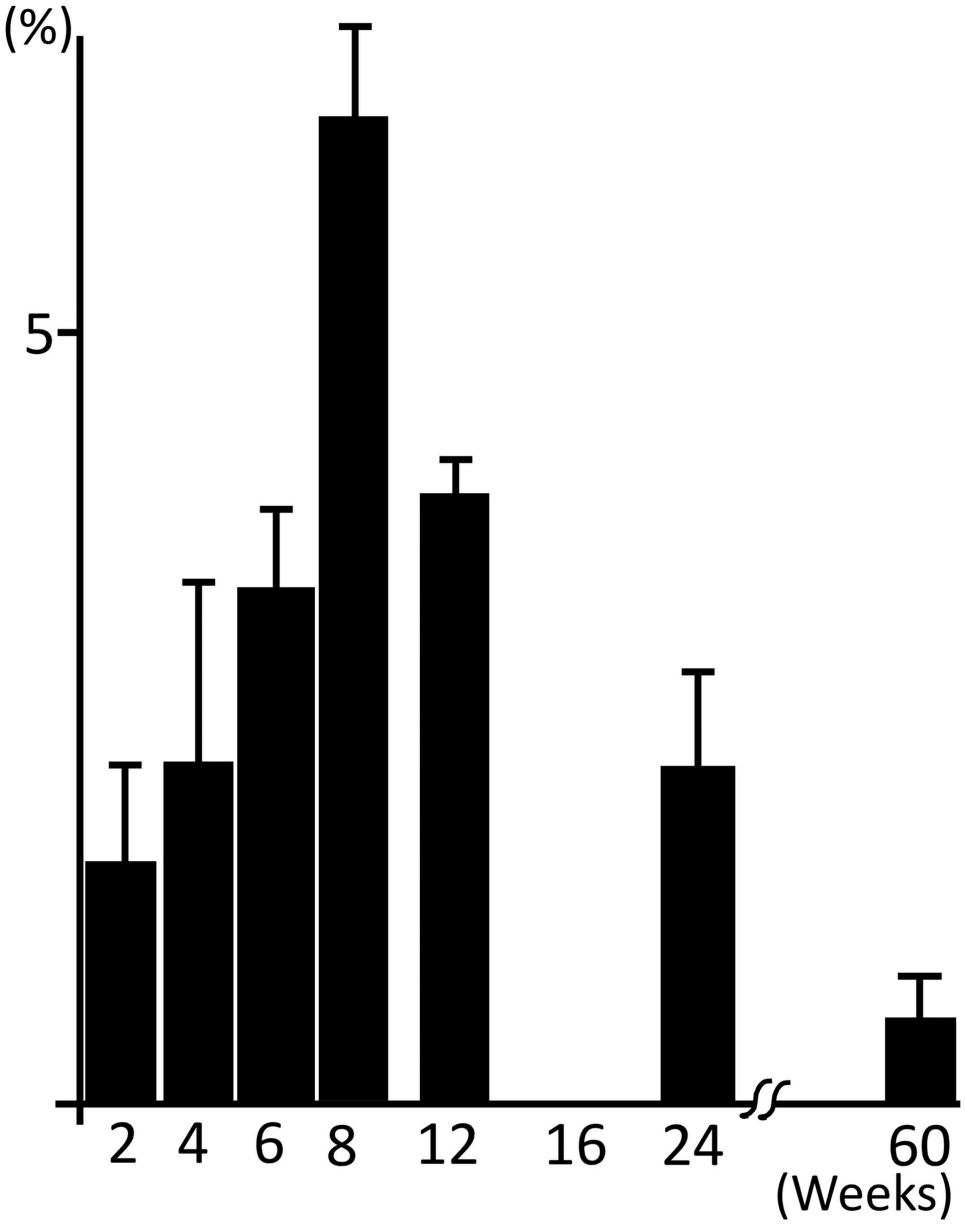
The numbers of DRG neurons covered with satellite cells showing strong GPR37L1 staining were counted, and the percentages for each postnatal week are shown. The peak occurred at 8 weeks.

The neurons covered by satellite cells with strong GPR37-IR and GPR37L1-IR were counted; the percentages of these neurons are shown in Fig 6. The number of neurons with satellite cells showing strong receptor IR increased after 2 weeks, peaked at 8 weeks, and then decreased (Fig 6).

### 3.5 Comparison of GPR37 and GPR37L1 by triple immunostaining

**Fig 7:**
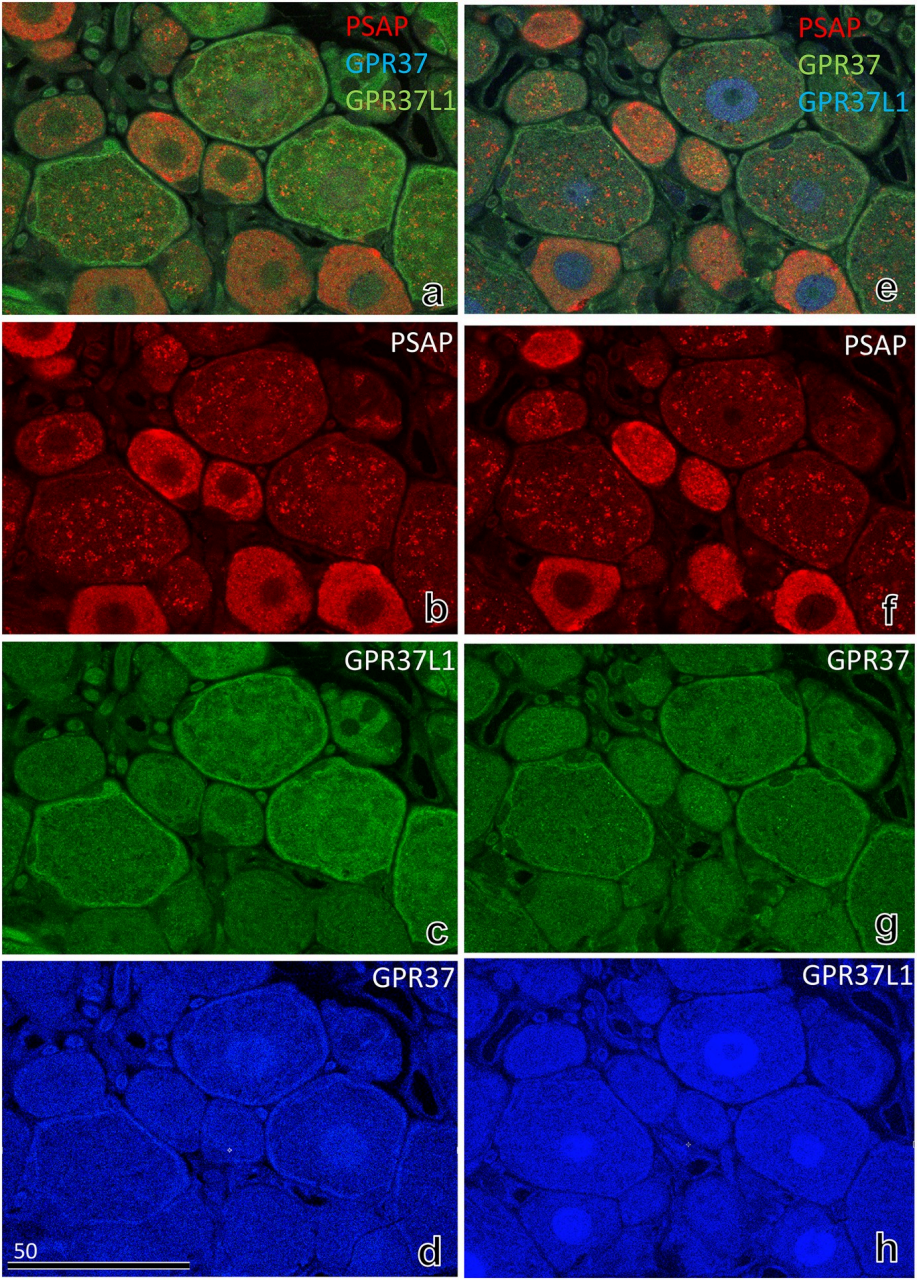
Immunofluorescence light micrographs of the rat DRG at 8 weeks, stained with antibodies against PSAP (red), GPR37L1 (green), and GPR37 (blue) (a–d). The neighbouring section was stained with antibodies against PSAP (red), GPR37 (green), and GPR37L1 (blue) (e–h). The intensity of GPR37L1-IR in satellite cells was stronger than that of GPR37-IR (c, h). The numbers on the bars indicate the scale in μm.

From the above results, GPR37-IR and GPR37L1-IR appeared to be distributed in the same satellite cells, and staining of GPR37L1-IR was more intense than PR37-IR. To compare the intensity of GPR37-IR with GPR37L1-IR, serial sections (Fig 7a and 7e) were stained with antibodies conjugated with converted colours: GPR37 (blue; Fig 7d)/GPR37L1 (green; Fig 7c) or GPR37 (green; Fig 7g)/GPR37L1 (blue; Fig 7h). Although the intensity of GPR37L1-IR was slightly stronger than that of GPR37-IR (Fig 7c–7h), IRs were similar despite the converted colours.

**Fig 8:**
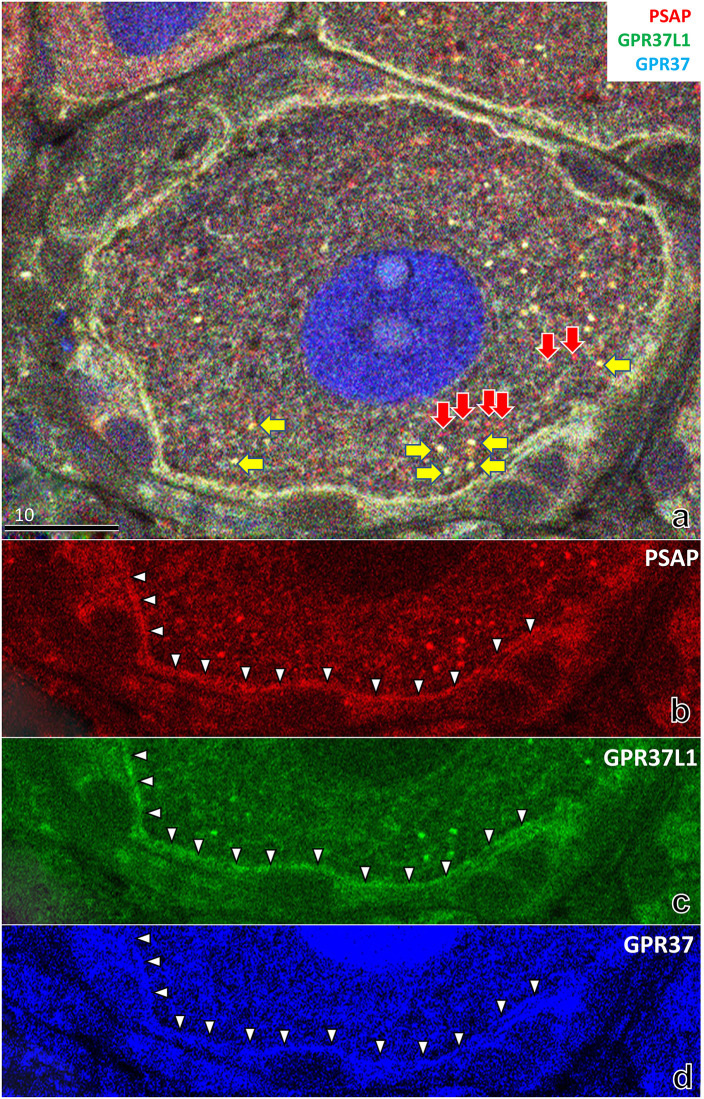
Higher magnification immunofluorescence light micrographs of the rat DRG at 8 weeks show the colocalization of PSAP and its receptors (GPR37, GPR37L1). In the lysosome-like structures in the neuronal cytoplasm, PSAP colocalized with GPR37L1 (yellow arrows), or existed in isolation (red arrows). Note that the lysosome with GPR37L1 is much bigger than the lysosome without GPR37L1(a, b). In the lysosome-like structures, colocalization of PSAP and GPR-37L1 was frequently observed (b, c), while PSAP did not colocalize with GPR-37 (b, d). In satellite cells, fine granular PSAP always colocalized with both GPR-37 and GPR-37L1, and accumulated near the boundary of neurons and satellite cells (arrowheads in b–d).

With higher magnification (Fig 8), colocalization of PSAP (IM1) and its receptors, GPR37 and GPR37L1, was more evident. In the cytoplasm of neurons, PSAP sometimes colocalized with its receptors (Fig 8a). In the lysosome-like structures, colocalization of PSAP and GPR-37L1 was frequently observed (Fig 8b and 8c), but PSAP did not colocalize with GPR-37 (Fig 8b and 8d). The lysosome-like structures that exhibited both PSAP-IR and GPR37L1-IR were much bigger than those with PSAP-IR only (Fig 8a and 8b). By contrast, in satellite cells, fine granular PSAP always colocalized with both GPR-37 and GPR-37L1, and accumulated near the boundary of neuron and satellite cells (Fig 8b–8d).

**Fig 9:**
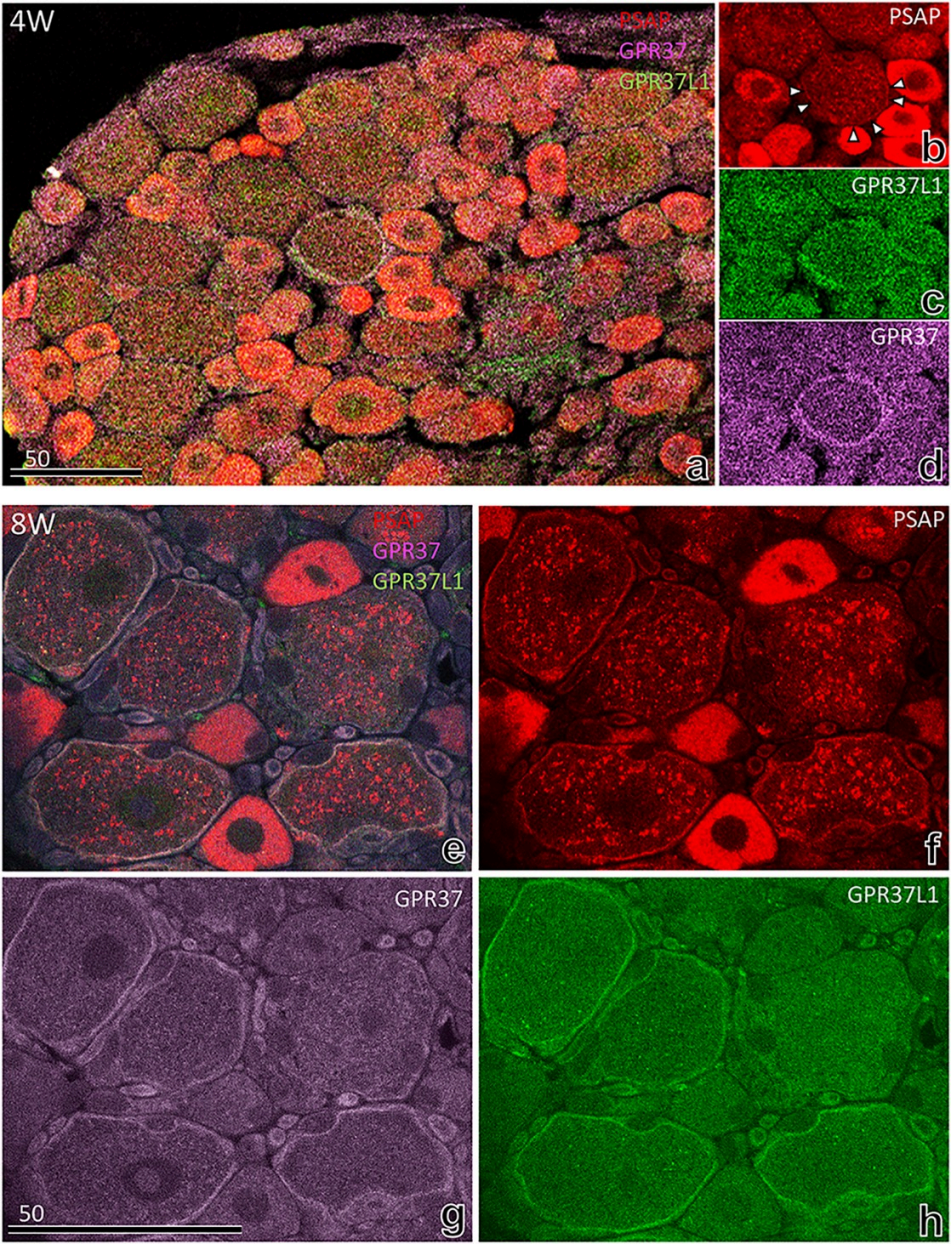
Immunofluorescence light micrographs stained with commercial antibodies against GPR37 (purple)/GPR37L1 (green) at 4 and 8 weeks after birth. The results were similar to those obtained with our autonomous antibodies; fine granular PSAP in satellite cells always colocalized with both GPR-37 and GPR-37L1 in the adult DRG (e–h); in young DRG neurons, PSAP did not always colocalize with receptors (a–d). In (b), only half of the satellite cells were outlined with fine granular PSAP (white arrowheads).

Furthermore, to verify the characteristic ring shape, sections were stained with commercial antibodies against GPR37 (purple)/GPR37L1 (green) at 4 and 8 weeks after birth (Fig 9); the results were consistent with those described above. Fine granular PSAP in satellite cells colocalized with both GPR-37 and GPR-37L1 in the adult DRG (Fig 9e–9h); in young DRG neurons, PSAP did not always colocalize with its receptors (Fig 9b).

### 3.6 Relationships of PSAP and its receptors with sortilin

**Fig 10:**
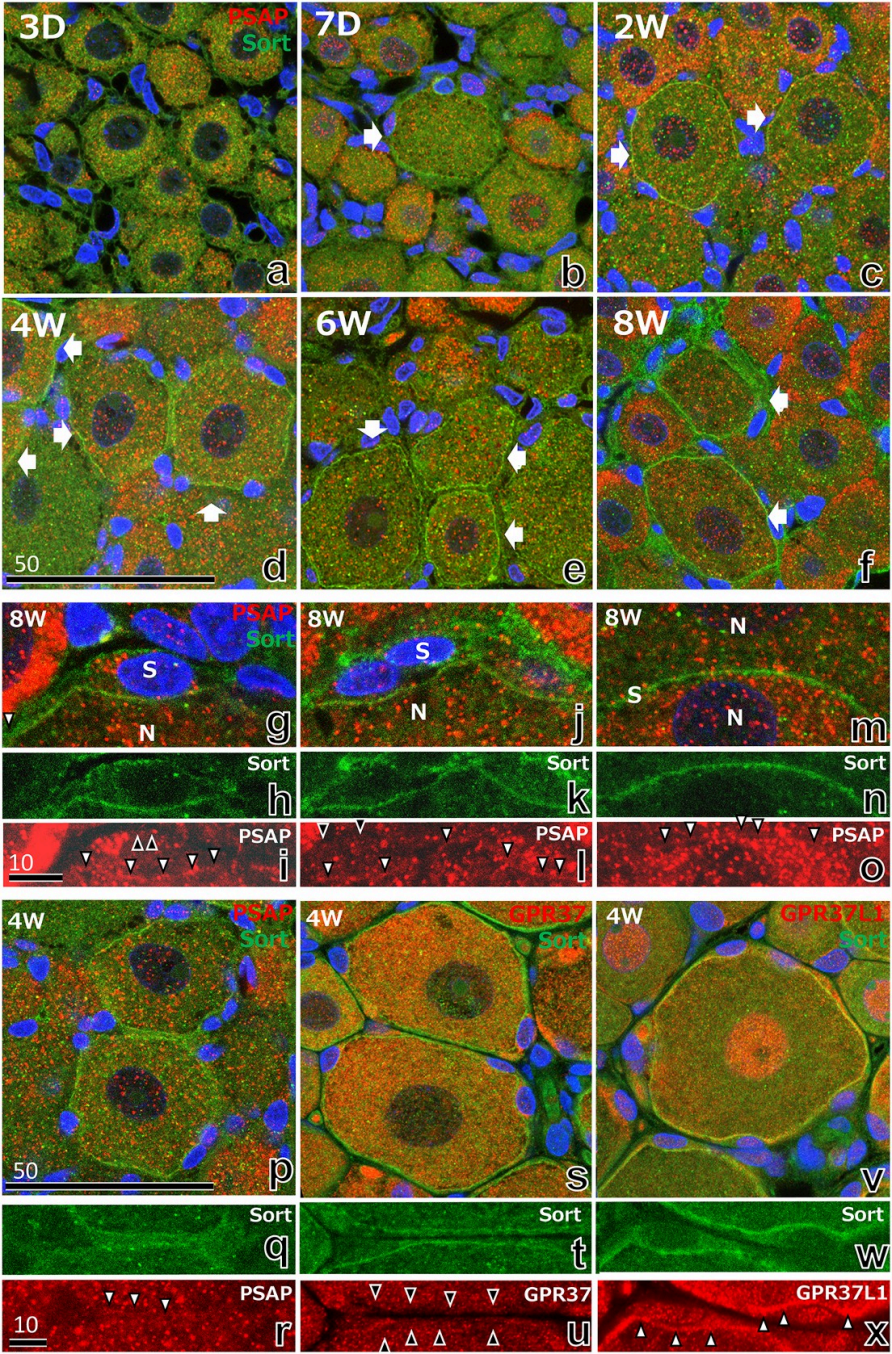
(a–f) Immunofluorescence light micrographs of the rat DRG from 3 days to 8 weeks after birth, stained with anti-PSAP (red), anti-sortilin (Solt, green), and DAPI. Weak sortilin-IR was first observed at postnatal day 7 in satellite cells (arrows in b). After postnatal week 2, the number of sortilin-positive cells increased, peaking at postnatal week 4 (arrows in d) and decreasing after postnatal week 8 (f). (g–o) Higher magnification immunofluorescence light micrographs of the rat DRG at 8 weeks after birth stained with antibodies against PSAP (red), sortilin (green), and DAPI (blue). (g, j) Both the outer and inner cell surface of satellite cells were clearly outlined by sortilin, while only the inner surface of satellite cells was outlined with fine granular PSAP (white arrowheads in i, l, and o), and not the outer cell surface (black arrowheads in i, l). (m) Rare case of a thin satellite cell (S) between two neurons (N), indicating clear colocalization of sortilin (n) and PSAP (o). (p–x) The relationships of PSAP and its receptors (GPR37, GPR37L1) with sortilin was studied by double immunofluorescence of the DRG at postnatal week 4. Sortilin-IR was localized in satellite cells, especially near the boundary to nerve cells (q, t, and w), while PSAP-IR (r) and GPR37-IR (u) were dispersed in the cytoplasm of satellite cells. Only GPR37L1 clearly colocalized with sortilin (w, x). The numbers on the bars indicate the scale in μm.

The relationship between PSAP and sortilin, the transporter of PSAP [36] was studied with double immunofluorescence of PSAP and sortilin (Fig 10a–10x). Weak sortilin-IR was observed at postnatal day 7 in satellite cells (Fig 10b). After postnatal week 2, the number of sortilin-positive cells increased (Fig 10c–10f), peaking at postnatal week 4 (Fig 10d) and decreasing thereafter until week 12.

Higher magnification immunofluorescence light micrographs of the rat DRG at week 8 (Fig 10g and 10j) showed that both the outer and inner surfaces of satellite cells were outlined clearly by sortilin (Fig 10h and 10k). On the other hand, fine granular PSAP outlined only the inner, and not the outer, surface of satellite cells (Fig 10i and 10l). Fig 9m shows a rare case of a thin satellite cell between two neurons, and indicates clear colocalization of sortilin (Fig 10n) and PSAP (Fig 10o) in the satellite cell.

The relationships of PSAP and its receptors with sortilin was studied in the DRG at postnatal week 4 (Fig 10p–10x). Sortilin-IR was localized in satellite cells, especially at the nerve cell boundary (Fig 10q, 10t and 10w), while PSAP-IR (Fig 10r) and GPR37-IR (Fig 10u) were dispersed in the cytoplasm of satellite cells. GPR37L1 clearly colocalized with sortilin (Fig 10w and 10x).

## 4. Discussion

To distinguish the distribution of PSAP from that of saposins, an anti-PSAP-specific antibody (IM-1) was generated against a synthetic oligopeptide corresponding to the proteolytic portion of PSAP (Fig 1). Although this epitope from the proteolytic portion of PSAP did not show good antigenicity, we nonetheless successfully generated a specific antibody after several trials. This antibody is essential for the study of PSAP, as previous anti-PSAP antibodies have been shown to react not only to PSAP, but also to some saposins.

The present study showed that PSAP exists primarily in the lysosomes of nerve cells and satellite cells in the DRG. We also report that PSAP mRNA exists primarily in nerve cells, but not in the satellite cells [35]. By contrast, PSAP receptors (GPR37/GPR37L1) mainly exist in satellite cells around nerve cells; notably, they accumulated near the boundary of neurons (Figs 8b–8d and 9). In some lysosome-like structures, clear colocalization of PSAP and GPR37L1 was observed (Fig 8b and 8c). Based on these results, we suggest that PSAP synthesized in nerve cells plays a role not only as a neurotrophic factor, but also as a gliotrophic factor in satellite cells. Liu et al. reported that glio- and neuro-protection conferred by PSAP is mediated by GPR37L1 and GPR37 [25].

The intensity and distribution of PSAP-IR in neurons varied greatly depending on the size of neurons (Figs 3b and 3g and 7b and 7f). PSAP-IR in larger neurons mainly existed in large lysosome-like structures; in smaller neurons, it was present in fine lysosome-like structures. Electron microscopy revealed that PSAP-IR was present mainly in larger lysosomes (Fig 4a and 4b); however, PSAP-IR was also present in fine lysosome-like structures in smaller neurons (Fig 4c and 4d).

In the lysosome-like structures, although colocalization of PSAP and GPR-37L1 was frequently observed (Fig 8b and 8c), PSAP did not colocalize with GPR-37 (Fig 8b and 8d). Note that the lysosome-like structures that exhibited both PSAP and GPR37L1 were larger and/or exhibited stronger fluorescence than lysosomes that showed PSAP expression only (Fig 8a and 8b). Zeng et al. reported that inactivation of the sortilin gene led to a significant decrease in PSAP in lysosomes [37]. Involvement of GPR37L1 in larger lysosomes may indicate a role of GPR37L1 in the lysosome trafficking of PSAP.

Satellite cells in the DRG have long been studied, and their function is thought to be to protect or provide metabolic support to DRG neurons. The number of satellite cells around a neuron increases as the neuron grows [38,39]. The neuron-satellite cell boundary also increases as the neuron grows, and shows a specific structure that includes perikaryal projections that increase the neuronal cell surface, similar to microvilli in the intestine [40,41]. Satellite and Schwann cells catalyse morphological transformation from immature bipolar to mature pseudo-unipolar DRG neurons *in vitro* [42] and *in vivo* [43], indicating cell-cell interaction, which determines the developmental fate and subsequent differentiation.

In satellite cells, fine granular PSAP always colocalized with both GPR-37 and GPR-37L1, which accumulated near the neuronal boundary (Figs 8b–8d and 9e–9h). While immunohistochemical analysis of PSAP distribution has been reported in lysosome-like structures [35], this is the first report of a clear line of granular PSAP. Colocalization of these molecules occurred in some satellite cells around one neuron, generating a characteristic ring shape around a neuron. Ordinarily, some satellite cells cover one neuron (Figs 8a and 10p, 10s and 10v). While these satellite cells are independent, gap junctions between them have been reported in the developing DRG [38,44]; these junctions may cause the synchronicity. Avraham et al. reported that the sensory neuron and its surrounding glial coat form a functional unit that orchestrates nerve repair [45].

**Fig 11:**
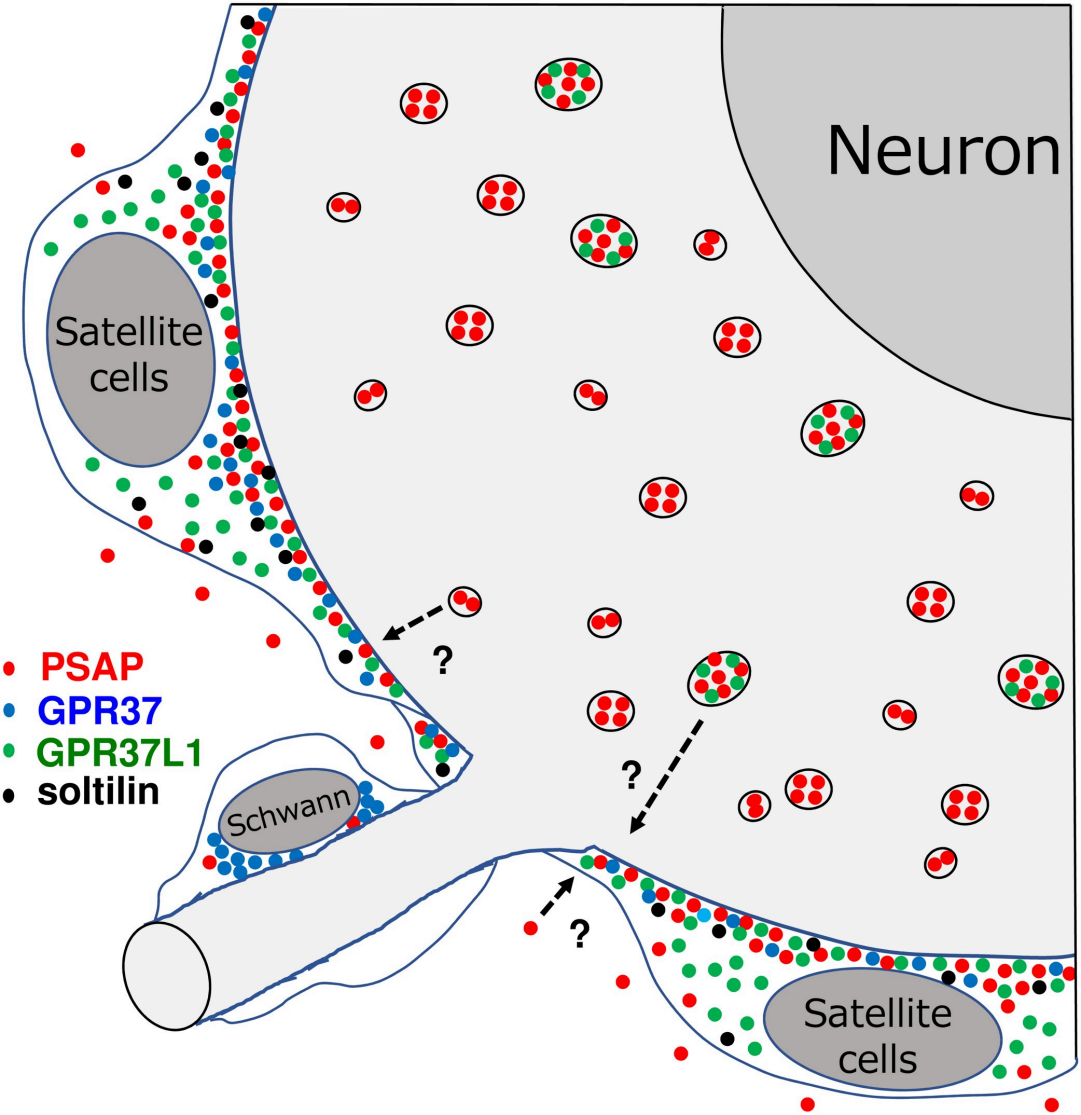
Schematic drawing of the interactions between neurons and satellite cells in the developing DRG. DRG neurons produced and contained PSAP in lysosomes with or without GPR37L1. At approximately 8 weeks, the satellite cells around larger neurons showed dispersed PSAP colocalized with both receptors near the neuron boundary. Sortilin, not shown in this figure, was observed at an earlier stage (2–4 weeks), and always colocalized with GPR37L1. In conclusion, DRG neurons produce PSAP, which may be delivered to satellite cells in a paracrine manner. It is also plausible that satellite cells take up PSAP from their surrounding extracellular space and transport PSAP to neurons. GPR37L1 and sortilin may transport PSAP to stimulate the proliferation or maturation of satellite cells.

The periods when these ring shapes appear is also important. They began to appear 2 weeks after birth; expression peaked at 8 weeks and decreased thereafter. In these periods, neurons develop quickly; the satellite cells covering neurons must proliferate, especially those around large, growing neurons, because there are more satellite cells around large compared with small neurons [39]. To stimulate the proliferation of satellite cells, neurons in the centre may send PSAP to them (Fig 11).

It is also possible that satellite cells take up PSAP from their surrounding extracellular space and transport PSAP to neurons (Fig 11). In this case, PSAP is uptaken with the help of sortilin, which accumulates in the outer cell membrane of satellite cells (Fig 10h and 10k), possibly with the help of PSAP receptors (Fig 10x). Accumulation of PSAP and its binding proteins (receptors and sortilin) in satellite cells, especially at the neuron boundary, suggests the stimulation of neurons by PSAP.

In either case, PSAP stimulate neurons and/or satellite cells with the help of its receptors and/or sortilin. By this characteristic phenomenon, PSAP, its receptors, and its transporter sortilin accumulate simultaneously at the neuron–satellite boundary, suggesting that PSAP plays a pivotal role in the developing peripheral nervous system.
